# Identification of diagnostic biomarkers for idiopathic pulmonary hypertension with metabolic syndrome by bioinformatics and machine learning

**DOI:** 10.1038/s41598-023-27435-4

**Published:** 2023-01-12

**Authors:** Wenzhang Lu, Jinbo Huang, Qin Shen, Fei Sun, Jun Li

**Affiliations:** 1 Department of Respiratory and Critical Care Medicine, Affiliated Hospital of Nantong University, Medical School of Nantong University, Nantong, 226001 China; 2 Department of Respiratory and Critical Care Medicine, Affiliated Hospital of Nantong University, Nantong, 226001 China

**Keywords:** Diagnostic markers, Mechanisms of disease, Metabolic syndrome, Hypertension

## Abstract

Idiopathic pulmonary hypertension (IPAH) is a condition that affects various tissues and organs and the metabolic and inflammatory systems. The most prevalent metabolic condition is metabolic syndrome (MS), which involves insulin resistance, dyslipidemia, and obesity. There may be a connection between IPAH and MS, based on a plethora of studies, although the underlying pathogenesis remains unclear. Through various bioinformatics analyses and machine learning algorithms, we identified 11 immune- and metabolism-related potential diagnostic genes (*EVI5L, RNASE2, PARP10, TMEM131, TNFRSF1B, BSDC1, ACOT2, SAC3D1, SLA2, P4HB,* and *PHF1*) for the diagnosis of IPAH and MS, and we herein supply a nomogram for the diagnosis of IPAH in MS patients. Additionally, we discovered IPAH's aberrant immune cells and discuss them here.

## Introduction

Pulmonary arterial hypertension (PAH) is a rare disorder characterized by the occlusion of arterioles in the lungs leading to marked increases in pulmonary vascular resistance^1^. There are many risk factors for PAH onset, including metabolic disorders, hyperlipidemia, obesity, insulin resistance, dysregulated vascular cell proliferation, abnormal cell metabolism, inflammation, and gene mutations^2^. Idiopathic PAH (IPAH) is an important type of PAH whose clinical symptoms lack specificity; patients mainly show symptoms related to progressive right heart dysfunction often induced by fatigue and display fatigue, dyspnea, chest tightness, chest pain, and syncope. IPAH is produced by multiple pathogenic factors, but the specific pathogenic mechanism has not been fully elucidated. Metabolic syndrome (MS) is triggered by a series of cardiovascular and metabolic risk factors that associate with one another. Its risk factors include metabolic abnormalities, hypertension, insulin resistance, glucose intolerance, central obesity, dyslipidemia, and inflammatory effects^3^. The pathogenesis of the two diseases has commonalities, and patients with MS are at higher risk of susceptibility to IPAH.

IPAH is considered a systemic disease and involves many organs and tissues as well as the inflammatory and metabolic pathways^4^. The roles of bone morphogenic protein receptor type 2 (BMPR2) and many of its downstream targets, such as peroxisome proliferator–activated receptor (PPAR)-γ and apolipoprotein E, in IPAH production induction through the metabolic pathway have been widely described, and PPAR-γ and apolipoprotein E are also related to a variety of pathological metabolic states^5^. Metabolic disorders have long been shown to be widespread in IPAH, and an increasing number of studies suggest the existence of a strong link between IPAH and MS; however, the two diseases have never been studied together. Thus, this study will explore the association between the two diseases and uncover common biomarkers for the diagnosis of disease.

Bioinformatics analysis helps us to mine the etiology of the disease, while gene microarray technology provides new ideas to explore the pathogenesis of IPAH and MS. In this study, we combined machine learning algorithms for bioinformatics analysis to identify candidate diagnostic genes and pathways shared by IPAH and MS from Gene Expression Omnibus datasets. This is also the first study to target the common biomarkers and related metabolic pathways of IPAH and MS, diagnostic gene expression validation was performed in another GEO dataset. Our study provides new insights for exploring the genetic etiology and combination treatment strategies for IPAH and METS comorbidity. In addition, we also investigated the infiltration of immune cells in IPAH. Materials and Methods.

### Data processing

**Figure 1:**
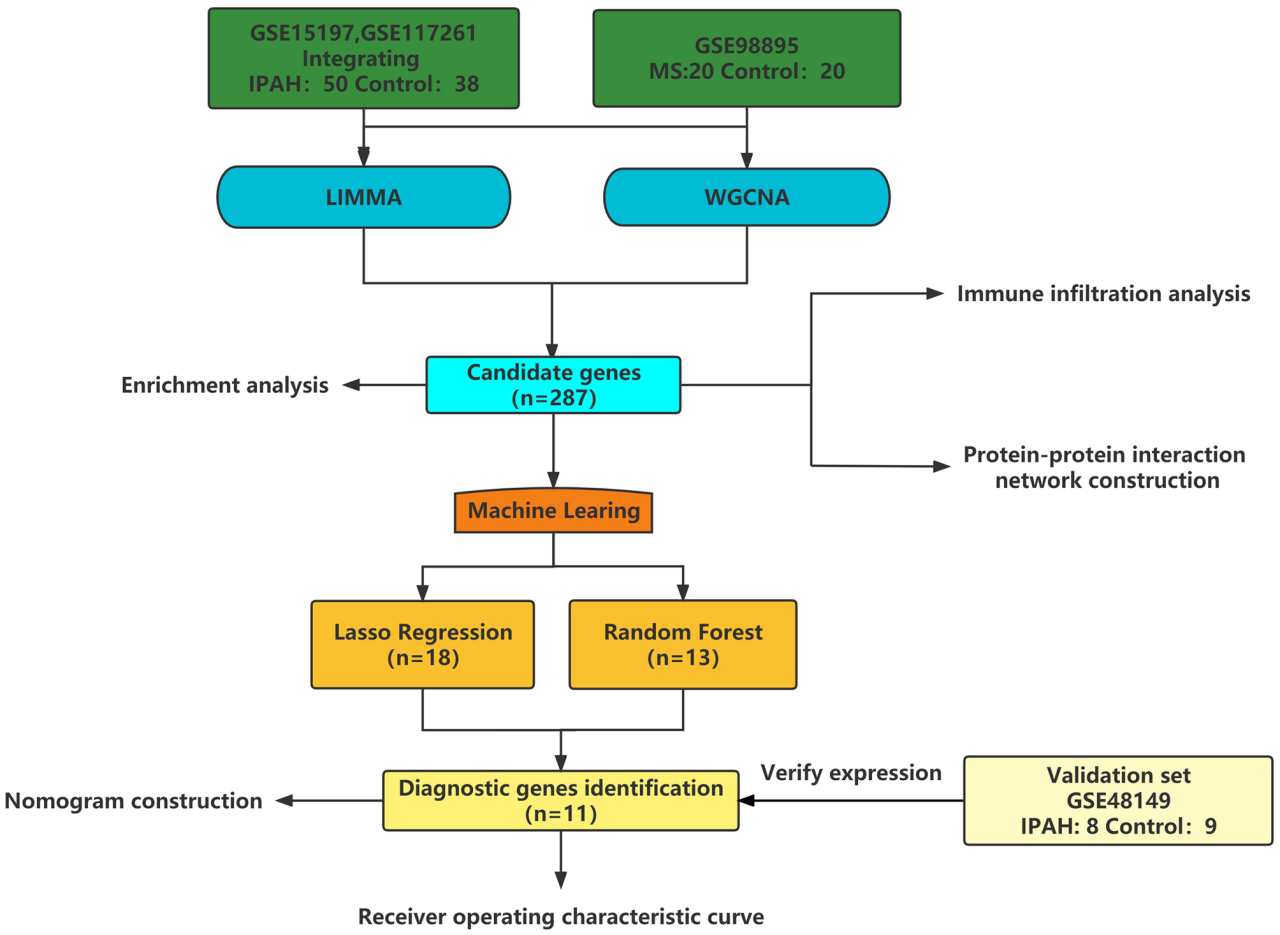
Flow chart of this study. Dawn by WPS office (version:11.1.0.11754).

A pair of datasets, GSE15197^6^ and GSE117261^7^, offering gene-expression data for IPAH patients and controls and a single dataset of MS patients, GSE98895^8^, were downloaded from the Gene Expression Omnibus database (https://www.ncbi.nlm.nih.gov/geo/)^9^. The GSE15197 series included samples from 13 control groups and 18 IPAH patient groups and the GSE117261 series included samples from 20 control groups and 32 IPAH patient groups, while the GSE98895 series included samples from 20 control groups and 20 MS patient groups. The IPAH database samples were obtained from human lung tissue and the MS database samples were obtained from patient peripheral blood. A single validation set, GSE48149^10^, containing 17 lung tissue samples from 8 IPAH patient groups and 9 normal controls, was also used. To avoid study errors, we excluded sex and age between patients and healthy controls in datasets. Details of these datasets are provided in Supplementary Table S1. The flowchart of this study is shown in Fig. 1, drawn by WPS office^11^ (Kingsoft, China, version: 11.1.0.11754).

### Screening for differentially expressed genes (DEGs)

First, both raw IPAH datasets were background-calibrated, normalized, and log2-transformed using the “affy” package in the R software program^12^. When multiple probes identified identical genes, the mean value was calculated to determine their expression. For the merging of multiple databases, we first merged the datasets using R, then used the method of Johnson, W. E to remove batch effects and finally obtained the matrix after batch effect removal^13^.

Limma is a differential expression screening method based on a generalized linear model^14^. We used the “limma” package in the R software program (version 3.40.6; R Foundation for Statistical Computing, Vienna, Austria, https://www.r-project.org/) for differential analysis to obtain the differential genes between the different comparison groups and the control groups. Finally, a |log2 fold change| value of > 0.5 (IPAH filtration) or 0.5 (MS filtration) and *p* < 0.05 were set as the criteria for identifying DEGs using the “limma” package.

### Weighted gene co-expression network analysis (WGCNA) and module gene selection

First, we counted the median absolute deviation for each gene using the gene expression pattern, and then removed the top 50% of genes with minimum median absolute deviation. The Good Samples Genes function within “WGCNA” (version 3.40.6; R Foundation for Statistical Computing, Vienna, Austria, https://www.r-project.org/) was used to remove unreserved genes^15^. Scale-free co-expression networks are then built. The Pearson correlation matrix and mean linkage method was used for all paired genotypes. Where β is the specified soft threshold parameter, and the power function is used to build the weighted adjacency matrix. Following the power selection, the adjacency relationship is transformed to a topological overlap matrix (TOM), and then the corresponding anisotropy (1-TOM) is computed. Furthermore, according to TOM’s measure of anisotropy, hierarchical clustering on the average chain is completed. The size of the graph corresponding to the smallest gene tree (gene cluster) is 100. We then set the sensitivity to be 3, combined modules with distance < 0.25, and ultimately obtained 10 modules of co-expression. Gray modules are collections of genes that are not assignable to any module.

### Functional enrichment analysis

After screening these DEGs and WCGNA signature biomarkers, we carried out Gene Ontology (GO) and analysis on the Kyoto Encyclopedia of Genomics (KEGG). Function enrichment analysis of gene sets was performed using an API in KEGG REST style (https://www.kegg.jp/kegg/rest/keggapi.html) software^16^. Gene annotations of the most recent KEGG pathway as a background were obtained and mapped to said background set; enrichment analysis was then performed using the R package “Profiler cluster” (version 3.14.3). For the purposes of this analysis, the smallest cohort size was 5 genes and the largest cohort size was 5 000 genes; *p* < 0.05 and an error-detection rate < 0.1 were considered to be statistically significant.

We also used the R package “org.Hs.eg” for GO annotations. We performed GO annotation of the gene using db (version 3.1.0) as a background for enrichment analysis and mapped the gene to said background set; we then re-ran the enrichment analysis using the R package “cluster Profiler” (version 3.14.3) to get the gene set enrichment result. The minimum set size was 5 genes and the maximum set size was 5, 000 genes; *p* < 0.05 and false discovery rate < 0.1 were considered statistically significant.

### Protein–protein interaction (PPI) network construction

We selected the STRING database (version 11.5; www.string-db.org) to mine the interactions between protein-coding genes^17^, and we established a PPI network. The required minimum interaction score was 0.4. Then, we used the Cytoscape software program (version 3.8.0; www.cytoscape.org/) to modify the images downloaded from STRING and identify important interaction genes using the M code plugin^18^.

### Machine learning algorithms to screen for candidate diagnostic genes

The random forest algorithm and the least absolute shrinkage and selection operator (LASSO) algorithm were used to screen for candidate diagnostic genes at the intersection of DEGs and WGCNA module genes. We used the “random forest” R package (version 3.40.6) to construct random forest classifiers to compare and rank features by importance^19^. Then, using the “glmnet” R package (version 3.40.6)^20^, we integrated gene-expression data for regression analysis using the LASSO Cox method. In addition, we also conducted fivefold cross-validation to obtain the optimal model. Genes selected by these two algorithms were sequentially included as candidate diagnostic genes.

### Nomogram construction and receiver operating characteristic (ROC) curve evaluation

To facilitate the clinical diagnosis of IPAH, we constructed a nomogram. Specifically, based on the aforementioned candidate diagnostic genes, we used the “rms” R package (version 3.40.6) to construct the nomogram^21^. “Points” indicates the score of candidate genes, and “Total Points” indicates the summation of all the scores of the genes above. The calibration curve of the nomogram was also constructed. The ROC curve was then established to evaluate the diagnostic value of the candidate genes, after which the area under the ROC curve (AUC) and 95% confidence interval (CI) values were calculated to quantify their impact.

### Statistical analysis

ROC curve establishment and the calculation of AUC and 95% CI values were completed using SPSS version 26.0 (IBM Corporation, Armonk, NY, USA). The proportions of different immune cells in the control and IPAH groups were compared by applying Student’s *t* test in GraphPad Prism version 8.3.0 (Graph Pad Software, San Diego, CA, USA). We considered *p* < 0.05 to be statistically significant.

### Comprehensive correlation analysis of infiltrating immune cells

IOBR is a computational tool for immune tumor biology studies^22^. CIBERSORT was selected based on our expression profiles using the “IOBR” R package (version 3.40.6)^23^, and 22 immune-infiltrating cell scores were calculated for each sample. The proportion of each type of immune cell in the different samples was visualized using Barplot. VioPlot was used to visualize the comparison of different proportions of types of immune cells between IPAH and controls. A heatmap depicting the correlation of the 22 scores of infiltrating immune cells was created with the “Corrplot” R package (version 3.40.6)^24^.

### Candidate diagnostic genes validation

As mentioned, we chose the GSE48149 dataset, which contains 17 lung tissue samples with 8 IPAH and 9 normal controls, as the validation set for this study. And we analyzed the expression of several candidate diagnostic genes in this dataset.

## Results

### DEGs

**Figure 2:**
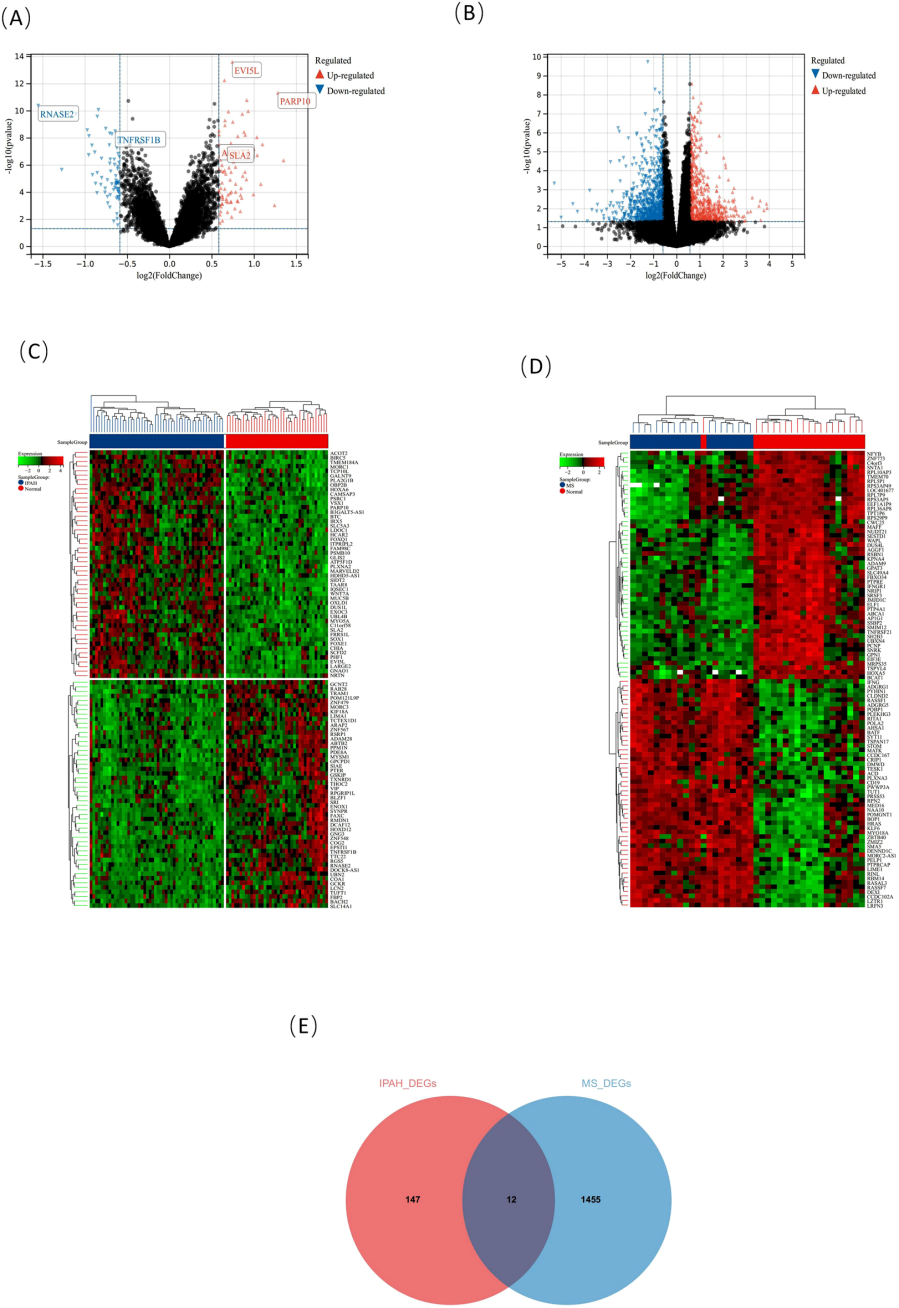
Volcano maps and heat maps of the IPAH and MS datasets. Drawn by R software program (version 3.40.6; R Foundation for Statistical Computing, Vienna, Austria, https://www.r-project.org/). (**A**) Volcano map of DEGs in IPAH dataset, |log2FC|> 0.5. (**B**) Volcano map of DEGs in MS dataset, |log2FC|> 0.5. Red represents up-regulated and blue represents down-regulated. (**C**) Heat map of DEGs in IPAH dataset. (**D**) Heat map of DEGs in MS dataset. Red and green indicate up-regulated and down-regulated DEGs, respectively. (**E**) Venn plot of overlapping IPAH and MS datasets DEGs.

A total of 159 DEGs were identified using the limma method in the IPAH combined database, of which 88 were elevated and 71 were downregulated. The heatmap and volcano map of the IPAH DEGs are shown in Fig. 2A,C. For the MS dataset, 1,467 DEGs (629 elevated and 838 downregulated) were selected (Fig. 2B,D). The intersection of the two groups of DEGs is shown in Fig. 2E.

### WGCNA and key module identification

**Figure 3:**
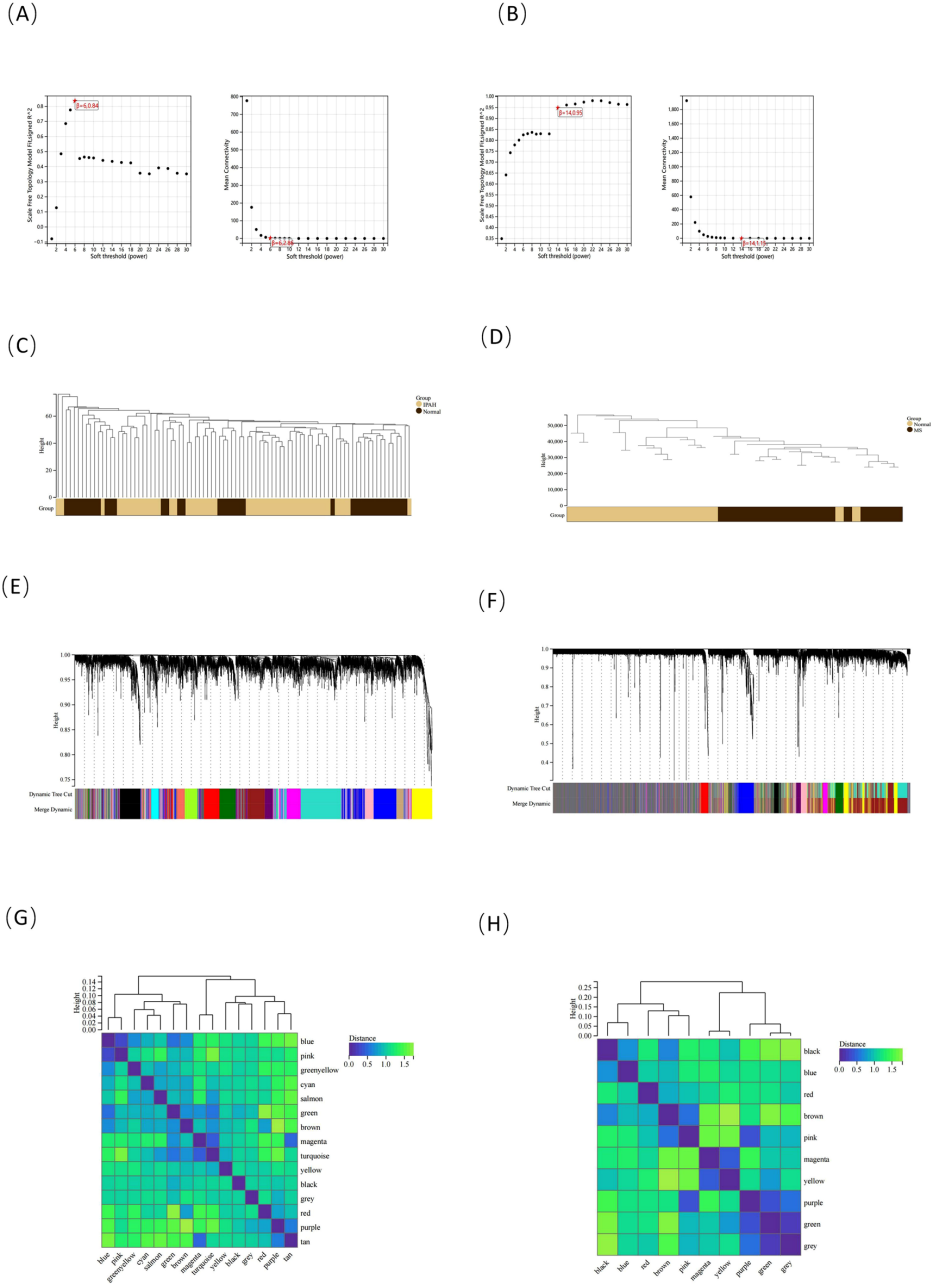
WGCNA in IPAH and MS. Drawn by R software program (version 3.40.6; R Foundation for Statistical Computing, Vienna, Austria, https://www.r-project.org/). (**A**) Analysis of scale independence and average connectivity in IPAH, select soft threshold β = 6. (**B**) Analysis of scale independence and average connectivity in MS, select soft threshold β = 14. (**C,D**) Sample clustering based on the expression level of patients in the IPAH/MS dataset. Outlier samples have been filtered. (**E,F**) Under the clustering tree, gene co-expression modules represented by different colors. (**G,H**) IPAH/MS Module feature vector clustering heatmap.

**Figure 4:**
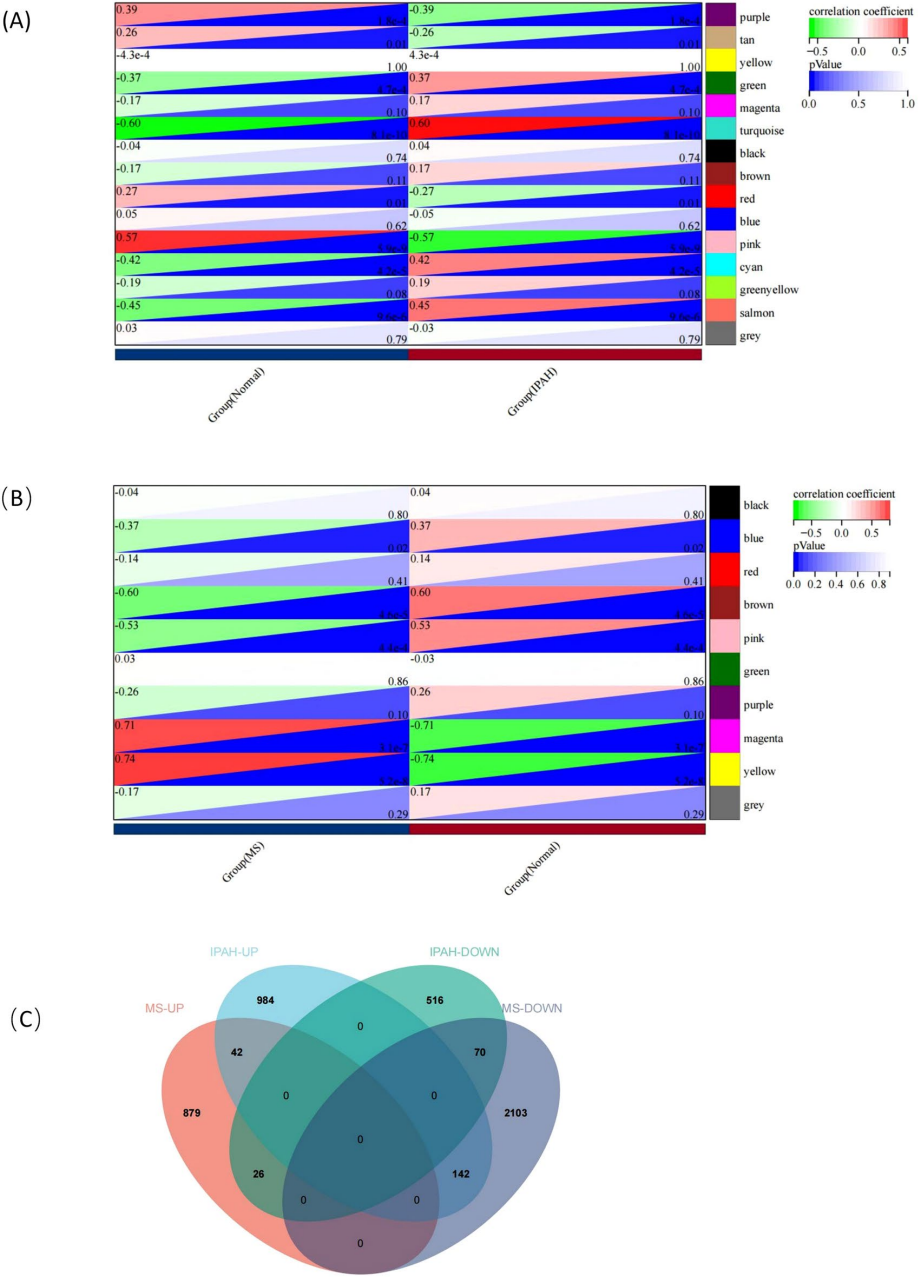
(**A**) Heat map of module correlation with IPAH. Turquoise 、cyan 、salmon modules had the strongest positive association with IPAH, pink and purple modules had the strongest negative correlation with IPAH. (**B**) Heat map of module correlation with MS. Magenta、 yellow modules had the strongest positive association with MS, brown and pink Modules had the strongest negative correlation with MS. (**C**) Venn plot of overlapping module genes for IPAH and MS. Figure 4 is drawn by R software program (version 3.40.6; R Foundation for Statistical Computing, Vienna, Austria, https://www.r-project.org/).

WGCNA was used to identify the most relevant modules in the IPAH and MS groups. In terms of IPAH, β = 6 was selected as the soft threshold based on the scale independence and average connectivity (Fig. 3A), while β = 14 was selected as the soft threshold for MS (Fig. 3B). Figure 3C,D depicts the cluster dendrogram of the IPAH/MS and controls. Based on this capability, in terms of IPAH, 15 gene co-expression modules were generated (Fig. 3E,G). A heatmap of module correlation with phenotypes is shown in Fig. 4A, where the turquoise, cyan, and salmon modules (1168 genes total) had the strongest positive associations with IPAH (R = 0.60, 0.42, and 0.45) and the pink and purple modules (612 genes total) had the strongest negative correlations with IPAH (R =  − 0.57 and − 0.39). In terms of MS, based on this capability, 10 gene co-expression modules were also generated (Fig. 3F,H), and a heatmap of module correlation with phenotype is shown in Fig. 4B, where the magenta and yellow modules (947 genes total) had the strongest positive associations with MS (R = 0.71 and 0.74) and the brown and pink modules (2315 genes total) had the strongest negative correlations with MS (R = − 0.60 and − 0.53). After WGCNA screening, we obtained 280 IPAH intersections with MS module genes (Fig. 4C).

### Functional enrichment analysis 

We identified a total of 12 intersection genes in the IPAH and MS DEGs, which included five genes that overlapped with those selected by WGCNA. To avoid omissions, we removed these overlapping genes and combined the DEG genes and module genes together as candidates for the following analysis.

**Figure 5:**
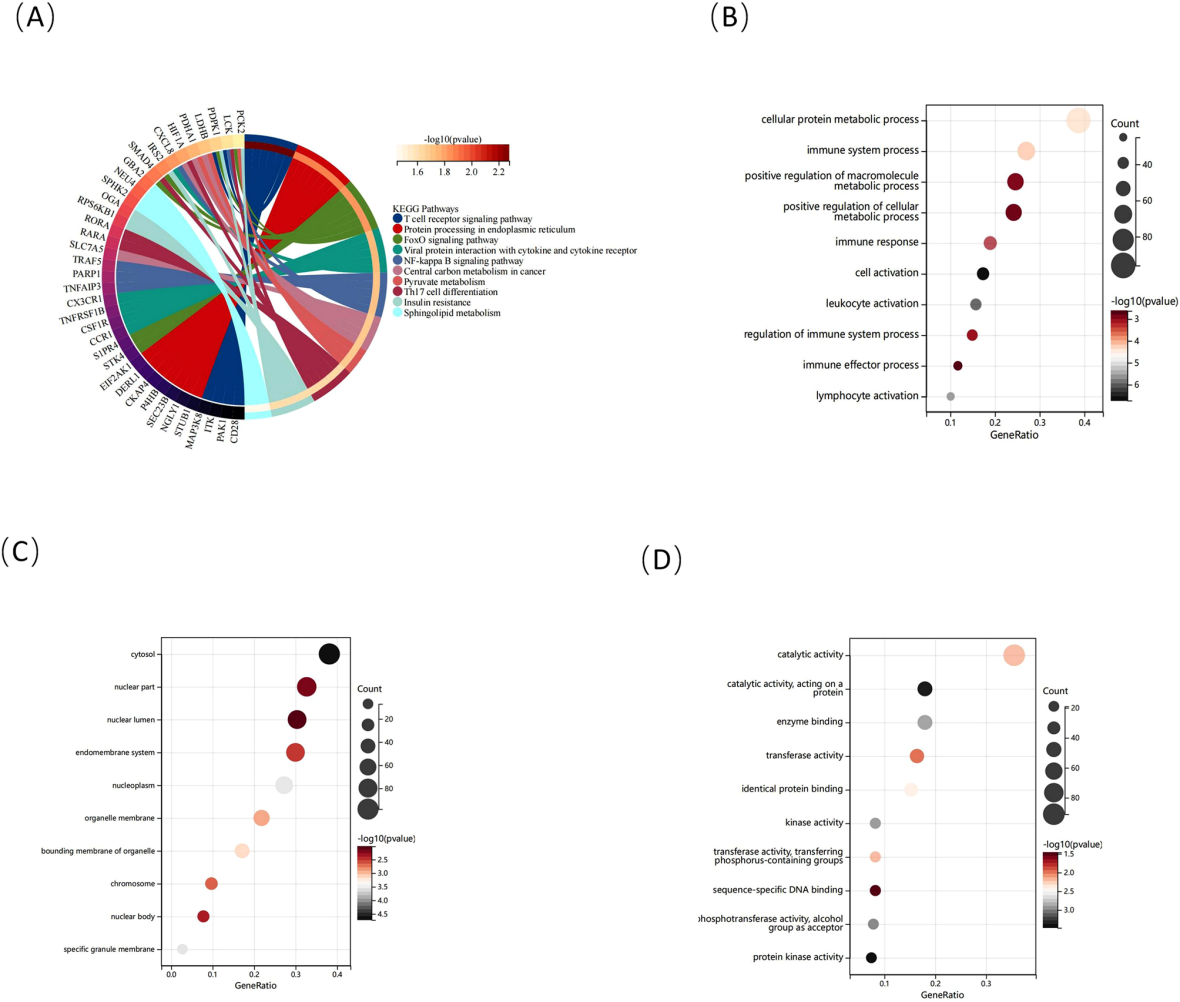
Enrichment analysis of candidate genes. (**A**) KEGG pathway analysis is shown in a circle diagram, with different colors representing different pathways. (**B–D**) Go analysis of candidate genes, including biological processes, cellular components, and molecular functions. Colored by P values, the X axis represents the proportion of enriched genes and the Y axis represents different results.

We further obtained 287 candidate genes. KEGG analysis revealed that these genes were primarily enriched in the “T-cell receptor signaling pathway” and “central carbon metabolism in cancer” pathway (Fig. 5A). GO analysis elucidated that the genes were mainly enriched under certain biological process terms, including “cellular protein metabolic process” and “immune system process” (Fig. 5B). With regard to cellular component ontology, these genes were mainly located in the “cytosol” and “nuclear part” categories (Fig. 5C). Molecular function analysis showed that “catalytic activity” and “catalytic activity acting on a protein” were the most significant categories among the genes (Fig. 5D). Enrichment analysis indicated that the candidate genes were mainly related to metabolism and the immune response and closely related to the pathogenesis of IPAH and MS, providing strong evidence for the subsequent analysis.

### PPI network construction

**Figure 6:**
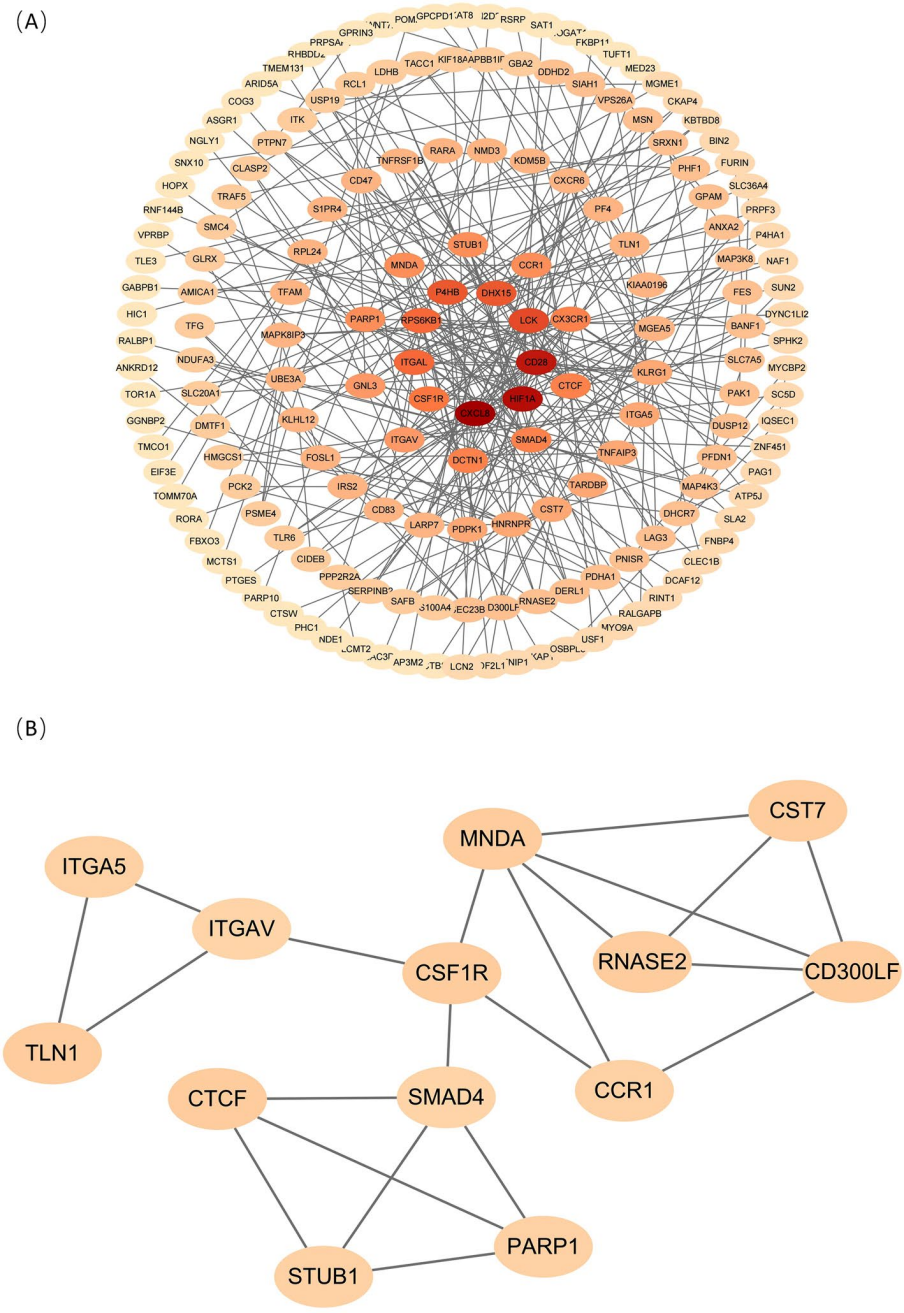
PPI network. Drawn by STRING database (version 11.5; www.string-db.org) and Cytoscape software (version 3.8.0; www.cytoscape.org/). (**A**) PPI network of candidate genes. Different gene colors indicate the degree of core of the gene in the PPI network, and the darker the color, the higher the degree of core. (**B**) Filter the most prominent modules by the MCODE plugin.

After confirming that the selected genes are closely related to immunity and metabolism, we constructed a PPI network to identify node genes. Figure 6A shows the PPI network, where the most active modules were visualized using the M code plugin for Cytoscape (Fig. 6B). 13 genes were identified as Hub genes, and functional enrichment was found to be mainly enriched in “Immune system process” and “Cell surface receptor signaling pathway”. This reveals that the Hub genes plays a central role more in the PPI network through the immune system. Specific information is presented at Supplementary Table S2.

### Identification of candidate diagnostic genes via machine learning

**Figure 7:**
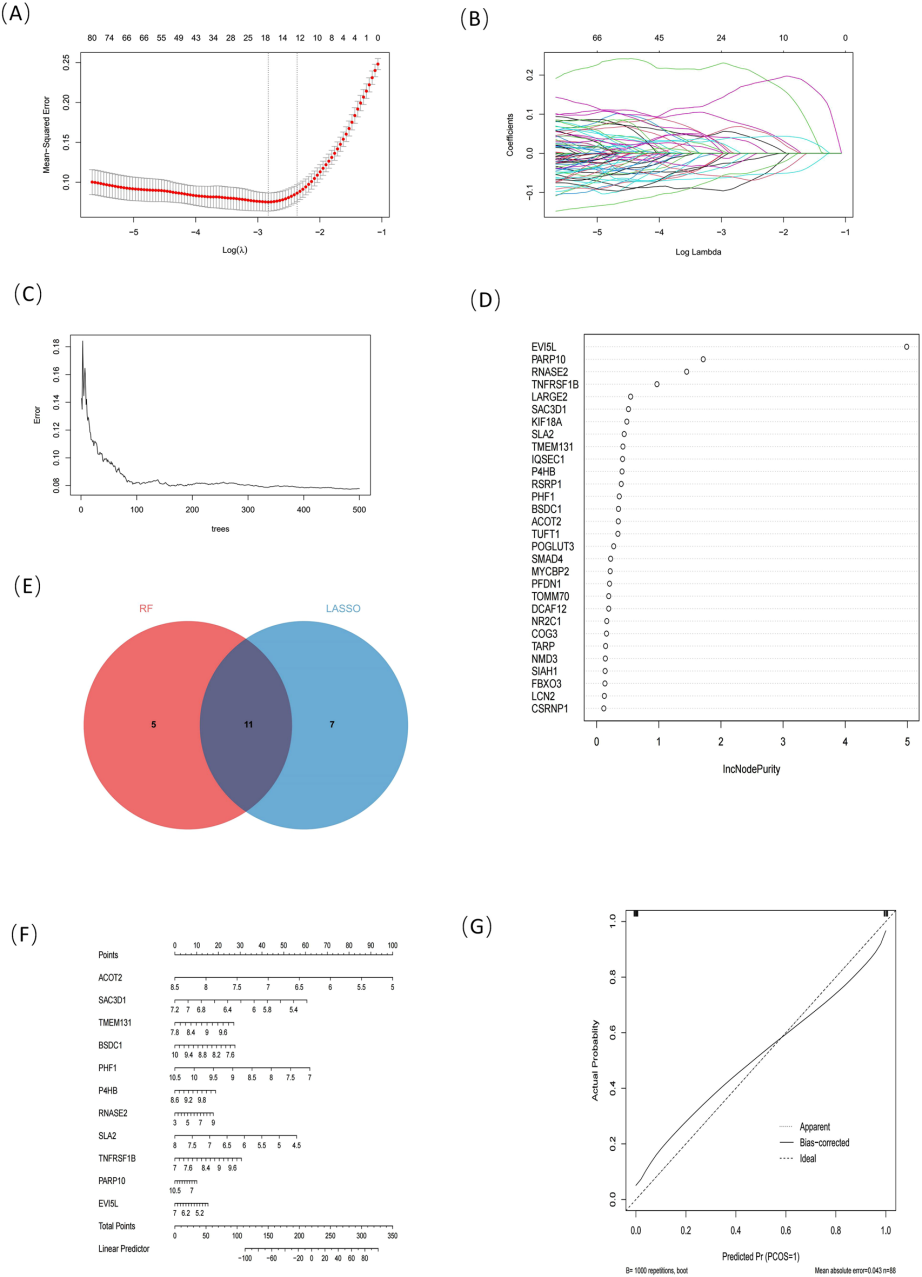
Machine learning. Drawn by R software program (version 3.40.6; R Foundation for Statistical Computing, Vienna, Austria, https://www.r-project.org/). (**A,B**) Genes are screened by the LASSO algorithm. In order to obtain the optimal model, the ten-fold cross-validation method is adopted. The lowest gene number n = 18 at the lowest point of the curve is best suited for LASSO. (**C,D**) Screen genes via random forest algorithm. The top 30 significant genes recognized from Random Forest. IncNodePurity rank the genes in accordance with their relative importance. (**E**) Venn plot of the intersection of two algorithms. (**F**) Nomogram for IPAH and MS diagnostics. (**G**) Nomogram's calibration curve, Close to the diagonal indicates high accuracy.

In this study, we applied LASSO regression and the random forest machine learning algorithm to filter candidate genes for nomogram construction and diagnostic value assessment. (Fig. 7A,B) shows that the LASSO regression algorithm identified 18 potential biomarker candidates and in the random forest the algorithm ranked genes by importance (Fig. 7C,D). With the aid of a Venn diagram (Fig. 7E), we show the intersection of the 16 most highly ranked genes from the random sample of the forest with the top 18 potential LASSO candidate genes and the top 11 genes (*EVI5L, RNASE2, PARP10, TMEM131, TNFRSF1B, BSDC1, ACOT2, SAC3D1, SLA2, P4HB*, and *PHF1*) may be regarded as having the highest diagnostic value.

### Diagnostic value assessment

**Figure 8:**
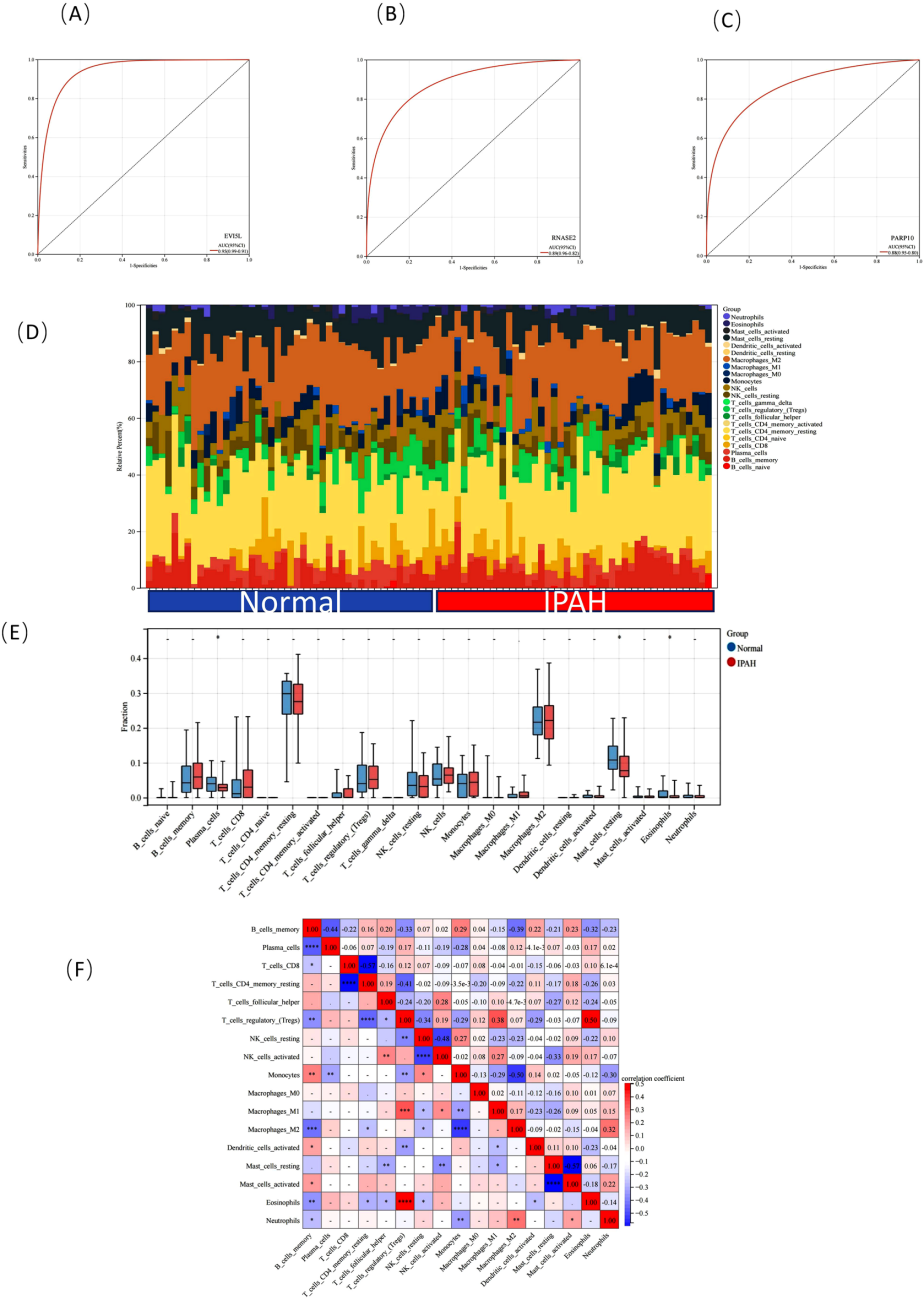
(**A–C**) ROC curve of part of the candidate diagnostic genes: (EVI5L(AUC = 0.95,95%CI 0.91–0.99), RNASE2(AUC = 0.89,95%CI 0.82–0.96), PARP10(AUC = 0.88,95%CI 0.80–0.95). (**D**) Twenty-two immune-infiltrating cell scores were calculated for each sample. Using Barplot to visualize the proportion of each type of immune cell in different samples. (**E**) Comparison of immune cells with different proportions between IPAH and control groups. (**F**) IPAH immune cell ratio-related heat map. Red represents a positive correlation and blue represents a negative correlation. Figure 8 is drawn by R software program (version 3.40.6; R Foundation for Statistical Computing, Vienna, Austria, https://www.r-project.org/).

A nomogram was constructed based on the 11 candidate diagnostic genes (Fig. 7F). ROC curves were also established to assess the diagnostic specificity and sensitivity of each gene and nomogram. The calibration curve of the nomogram is shown in Fig. 7G, and the closer the bias-corrected line is to the diagonal, the greater the diagnostic value of the nomogram. We calculated the AUC and 95% CI values for each project. Three genes with the highest AUC values were selected for display in Fig. 8A–C, as follows: *EVI5L* (AUC = 0.95, 95% CI 0.91–0.99), *RNASE2* (AUC = 0.89, 95% CI 0.82–0.96), and *PARP10* (AUC = 0.88, 95% CI 0.80–0.95). The AUC values for all genes are shown in Supplementary Table S3. All candidate genes had diagnostic value for IPAH with MS; other genes had AUC values that fluctuated from 0.76 to 0.88.

### Immune cell infiltration analysis

Based on the results of previous enrichment analyses, it can be inferred that the common genes of IPAH and MS are enriched in metabolic and immune-related pathways and can be used as a diagnostic means for the potential biomarker abundance of IPAH. Therefore, the relevant mechanisms can be better explored by the immune cell infiltration analysis of IPAH. For the IPAH and control groups, the proportion of 22 immune cells in each sample is shown in Fig. 8D. Figure 8E shows that IPAH patients have higher levels of memory B-cells, CD8 T-cells, follicular helper T-cells, monocytes, and M1 and M2 macrophages and lower levels of plasma cells, memory resting CD4 T-cells, regulatory T-cells (Tregs), resting natural killer (NK) cells, NK cells, resting mast cells, and eosinophils. The correlation of 22 types of immune cells revealed that Tregs were positively associated with eosinophils (r = 0.50) and macrophages M1 (r = 0.38), whereas CD8 T-cells were negatively related to memory resting CD4 T-cells (r = − 0.57) (Fig. 8F). In IPAH patients, various immune cell infiltrates differed, which may be a potential regulatory point for IPAH treatment.

### Candidate diagnostic genes validation

**Figure 9:**
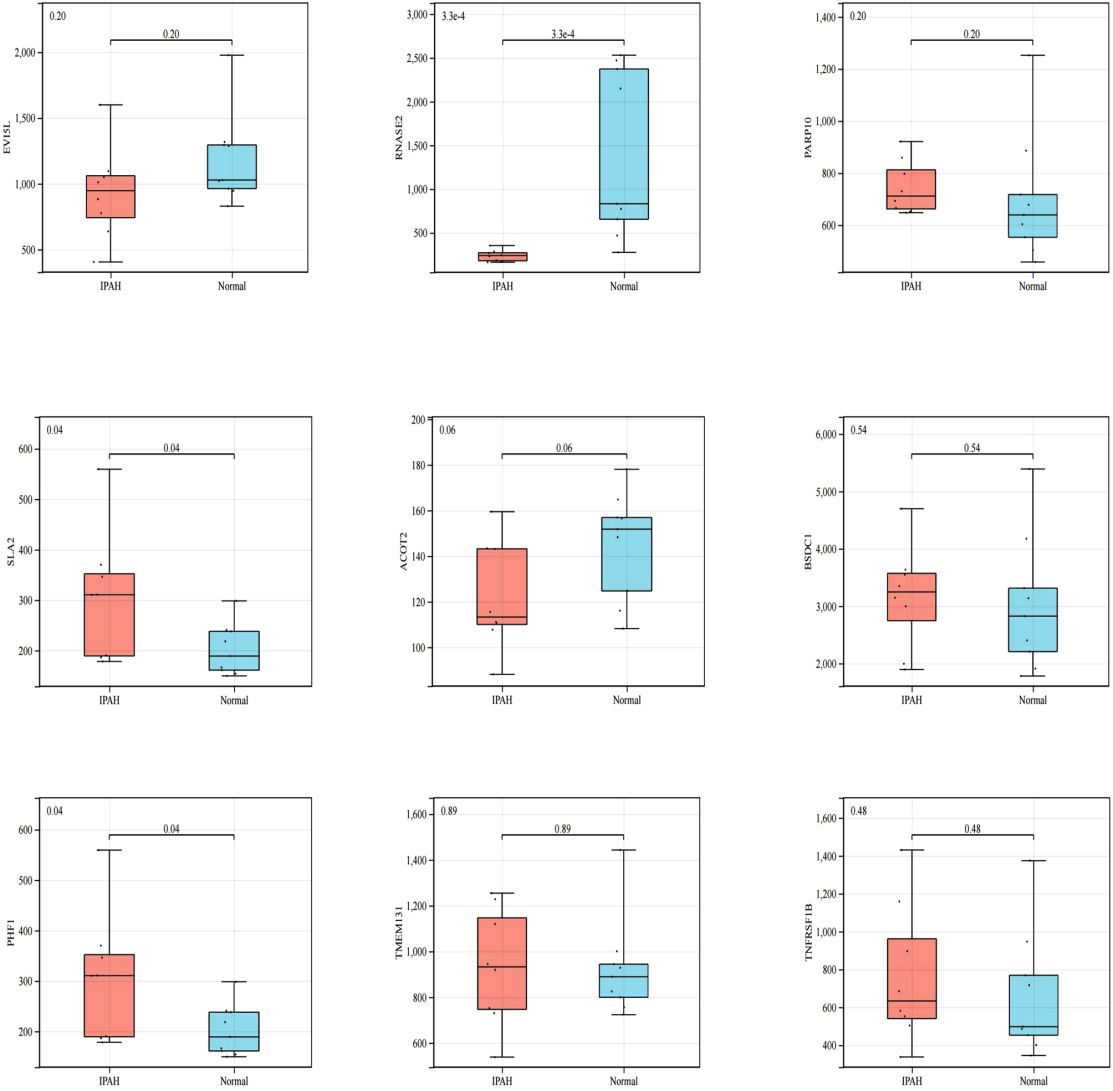
Lung tissue IPAH dataset GSE48149 verifies the expression of candidate diagnostic genes.

To validate candidate diagnostic gene expression in IPAH patients, we analyzed the expression of the differential genes in the validation set, and the correlation results are shown in Fig. 9. The results showed that the candidate diagnostic genes were differentially expressed in lung tissue dataset of IPAH patients, with the difference in *RNASE2* being the most significant.

## Discussion

The etiology of IPAH is unknown, yet the disease places a great physical, mental, and economic burden on patients. Existing studies have identified a proportion of new biomarkers able to facilitate the diagnosis of IPAH. Immune-infiltration studies of IPAH have been initially reported previously, but this investigation is the first to combine IPAH with MS. Meanwhile, the identification of candidate diagnostic genes has not been considered in the diagnosis of IPAH. We used a series of integrated bioinformatics analyses and machine learning methods to identify common pathways and shared candidate diagnostic genes for IPAH and MS. To avoid errors, we combined the DEGs and WGCNA module genes to identify a total of 287 shared candidate genes. Enrichment analysis indicated that these candidate genes are associated with immune- and metabolism-related signaling pathways. Next, we applied a machine learning approach to further screen for key genes. The crossover results of random forest and LASSO analyses were considered shared candidate diagnostic genes for IPAH and MS, and we further validated the diagnostic effect of each shared candidate diagnostic genes. In particular, *EVI5L*, *RNASE2*, and *PARP10* have great diagnostic value and high AUC values.

IPAH is a rare disease characterized by increased pulmonary vascular resistance. In this study, we selected two datasets with IPAH lung tissue, which were more representative of gene expression in IPAH patients than peripheral blood gene sequencing, as analytical samples. We then verified results above using the data and found that the identified candidate diagnostic genes were equally differentially expressed in another IPAH lung tissue dataset. Therefore, we could infer that the discovered candidate diagnostic genes can detect hidden IPAH through peripheral blood examinations of MS patients, which is an insanely simple and economical operation and avoids an invasive examination by right heart catheterization.

Ultimately, we identified 11 key candidate genes (*EVI5L, RNASE2, PARP10, TMEM131, TNFRSF1B, BSDC1, ACOT2, SAC3D1, SLA2, P4HB,* and *PHF1*), and a nomogram to diagnose IPAH in MS patients showing high diagnostic value was also established.

*EVI5L* belongs to a small subfamily of TRE-2/BUB2/CDC16 domain proteins and is a byproduct of *EVI5*. *EVI5L* has about a 70% similar identity to *Evi5*. Due to the few existing reports about *EVI5L*, however, we mainly analyzed *EVI5. EVI5* has different regulatory roles in cell cycle progression, cytokinesis, and cell membrane trafficking. In tumors, *EVI5* expression is dysregulated in multiple cancer types, such as non–small-cell lung cancer, laryngeal cancer, and hepatocellular carcinoma, and *EVI5* is therefore considered potential oncogenes and cell-cycle regulators^25–27^. *EVI5* is also a risk factor for multiple sclerosis^28^. Multiple sclerosis is a fairly common autoimmune demyelinating disease; *EVI5L* may therefore play an important role in cellular immunity as an immune-related gene. However, the mechanism of *EVI5L* in IPAH and MS requires further investigation.

*RNASE2* is an eosinophil-derived neurotoxin (EDN/RNase2) and an endolysosomal ribonuclease that acts synergistically to release uridine from oligonucleotides. *RNASE2* activates human toll-like receptor 8 (TLR8), whereas TLR8 activation induces a potent T helper-1 cell response critical for defense against intracellular pathogens. This suggests that *RNASE2* plays an important role in the immune system^29^. As an immune-related molecule, *RNASE2* is a biomarker of various immune system diseases, including chronic myelogenous leukemia, systemic lupus erythematosus, rheumatoid arthritis, and multiple myeloma^30–33^. In terms of cancer, *RNASE2* promotes the malignant progression of glioma through the PI3K/Akt signaling pathway^34^. It is also an immune-related biomarker used for evaluating the prognosis of gastric and kidney cancers^35,36^. In the respiratory system, *RNASE2* affects the eosinophil-specific protein levels of the asthma family and plays a key role in allergic reactions that trigger asthma^37^. Previous bioinformatics studies have indicated that *RNASE2* is overexpressed in IPAH and is a biomarker of IPAH^38^. However, existing research still does not clearly define the major role of *RNASE2* in IPAH. In this study, we found that *RNASE2* is a common immune- and metabolism-related biomarker for both MS and IPAH, which suggests that *RNASE2* may be responsible for the development of metabolic disorders in both diseases, proving it has an important potential role in diagnosing MS patients with IPAH.

*PARP10*, alternatively known as *ARTD10*, is a PARP protein family member that performs mono-ADP-ribosylation of target proteins^39^. *PARP10* is a metabolic regulator that plays an important role in lipid metabolism. Silencing of *PARP10* induces mitochondrial oxidation and AMPK activity. *PARP10* is involved in regulating cellular autophagy in cellular models; in a cell cancer model, loss of *PARP10* induces fatty acid oxidation^40^. *PARP10* is commonly expressed in human tissues, especially in the liver and spleen. The secretion of apolipoprotein B in the liver is dependent on *PARP10*, and *PARP10* silencing reduces apolipoprotein B expression in human hepatocytes^41^. Therefore, the expression of *PARP10* may affect very-low-density, intermediate-density, and low-density lipoprotein levels, and *PARP10* is closely related to lipid metabolism. *PARP10* is also involved in the inflammatory response and tumor development, being overexpressed in the majority of human tumors, including breast and ovarian tumors, oral squamous cell carcinoma, colorectal carcinoma, and hepatocellular carcinoma, and *PARP10* also plays a role in promoting the proliferation of related tumors^42–45^. In addition, *PARP10* is required for anti-DNA damage, and *PARP10* gene knockout causes cellular hypersensitivity to DNA damage and a DNA replication defect^46^. We determined that the crossover genes of IPAH and MS are mainly enriched in metabolic and immune pathways and found that *PARP10*, as a metabolic regulator, plays an important role in the development and development of both diseases. Our study has demonstrated that the overexpression of *PARP10* in patients with IPAH with MS may be a vital metabolic-related biomarker in patients and has high diagnostic value.

Metabolic disorders are an important pathogenesis of PAH, and drug-targeted treatment of a patient’s pathological metabolic state for the treatment of increased pulmonary artery pressure is an area actively being studied by many researchers. Animal model tests found that the hypoglycemic drug metformin improved endothelial function in PAH and significantly increased the survival of PAH rats^47^. The results of a clinical trial also confirmed that biguanide, a hypoglycemic drug administered orally, significantly improved the right ventricular fraction area of PAH patients, with a good therapeutic effect^48^. Legchenko et al. found that the PPAR-γ agonist pioglitazone reversed pulmonary hypertension through fatty acid oxidation mainly associated with lipid metabolism and disturbed mitochondrial morphology/function in right ventricular failure and pulmonary vascular hypertension^49^. The sodium–glucose cotransporter 2 (SGLT2) inhibitor englizin enhanced urinary glucose excretion and reduced cardiovascular events and mortality in patients with type 2 diabetes. In their study^50^, found that SGLT2 reduced mortality in MCT-induced PAH rats and reduced maladaptive lung remodeling.

Inflammation is a critical component of all subtypes of PAH, activated immune cells secreted more cytokines, such as tumor necrosis factor-α and interleukins, can be found in the sera of patients at levels that positively correlate with the severity of disease in PAH^51^. Many circulating immune cells (e.g., macrophages, monocytes, mast cells, dendritic cells, and T-cells) have been shown to be activated in the spleen and lung in PAH, and a large number are recruited to or activated within the pulmonary circulation. They regulate pulmonary artery cell function and differentiation status in a paracrine fashion. The types of immune cells involved in PAH can become highly glycolytic on activation, suggesting that these cells might also be responsive to altered metabolic therapies and other factors^51^. Previous exploration of lung tissue biopsy samples from IPAH patients revealed perivascular inflammatory cell infiltration of T-cells, B-cells, and macrophages^52,53^. Austin et al. further found that CD8 T-cells in the lung tissue of IPAH patients were significantly increased in number and the inflammation caused by abnormal immune function and loss of autoimmunity was related to the pathophysiology of IPAH^54^. According to our results, IPAH patients have higher levels of memory B-cells, CD8 T-cells, follicular helper T-cells, monocytes, and M1 and M2 macrophages and lower levels of plasma cells, memory resting CD4 T-cells, Tregs, resting NK cells, NK cells, resting mast cells, and eosinophils. Our results are consistent with those of previous studies. Therefore, exploring the immune and metabolism mechanisms of IPAH could clearly pave the way for the diagnosis and treatment of IPAH. Above all, considering metabolic disorders and autoimmunity is crucial in exploring the pathophysiology of IPAH and mining therapeutics. Metabolic syndrome is a clinical feature mainly characterized by metabolic disorders. The two diseases are closely linked, and comprehensive analysis of the common biomarkers of these diseases can help with the early detection of hidden increased pulmonary vascular resistance in patients with MS, with timely medical intervention enabling greater avoidance of serious consequences.

Recent years, it has become a trend for medical scientists to use bioinformatics technology, machine learning algorithms and deep learning methods to solve related medical problems, and there are countless related literatures. Scientists have made some advanced computational models for analyzing existing lncRNA-disease associations and predicting potential human lnc RNAs for disease-disease associations, which can be effectively used to identify disease-associated lnc RNAs on a large scale and select the most promising disease-associated lnc RNAs for experimental validation^55^. There are also models based on network algorithms and models based on machine learning to predict new Circular RNAs-computational models for disease correlation^56^. While traditional biological experiments typically require a lot of time and money to study the differences in the concentration of certain metabolites in patients and those in healthy people, a new deep learning algorithm named as Graph Convolutional Network with Graph Attention Network (GCNAT) can predict potential associations of disease-associated metabolites^57^. Advanced model design has become more frequent in recent years, particularly in the form of reasonably combing multiple algorithms, a process known as model fusion. Combination of multiple algorithms to improve model performance and enhance predictive power has become the hottest trend^58^. Our research combines two machine learning algorithms to greatly enhance the predictive ability of IPAH and MS comorbid diagnosis genes, with high confidence.

## Limitations

Our study has several limitations. Although we pooled a pair of IPAH datasets, the total number of samples enrolled in this study remained limited. Although the identified candidate diagnostic genes were mainly enriched in regulating immune and metabolism pathways, the interactions between said candidate diagnostic genes and dysregulated immune cells are still worth further studying.

## Conclusion

To our knowledge, this is the first study to document diagnostic genes jointly associated with IPAH and MS. We identified a total of 11 immune- and metabolism-related candidate diagnostic genes (*EVI5L, RNASE2, PARP10, TMEM131, TNFRSF1B, BSDC1, ACOT2, SAC3D1, SLA2, P4HB,* and *PHF1*) through various bioinformatics analyses and machine learning algorithms, then provided a nomogram for the diagnosis of MS combined with IPAH. We also pointed out that a proportion of IPAH immune cells are dysregulated. Finally, differences in diagnostic gene expression were validated using lung tissue data from IPAH patients in GSE48149 database.

## Supplementary Information

Supplementary Information 1.

Supplementary Information 2.

Supplementary Table 1.

Supplementary Table 2.

Supplementary Table 3.

## Data Availability

The GSE117261, GSE15197, GSE98895, and GSE48149 microarray datasets used in this study were downloaded from the Gene Expression Omnibus database (https://www.ncbi.nlm.nih.gov/geo/query/acc.cgi?acc=GSE117261, https://www.ncbi.nlm.nih.gov/geo/query/acc.cgi?acc=GSE15197, https://www.ncbi.nlm.nih.gov/geo/query/acc.cgi?acc=GSE98895 and https://www.ncbi.nlm.nih.gov/geo/query/acc.cgi?acc=GSE48149).
